# Genetic evaluation of the breeding population of a valuable reforestation conifer *Platycladus orientalis* (Cupressaceae)

**DOI:** 10.1038/srep34821

**Published:** 2016-10-10

**Authors:** Yuqing Jin, Yongpeng Ma, Shun Wang, Xian-Ge Hu, Li-Sha Huang, Yue Li, Xiao-Ru Wang, Jian-Feng Mao

**Affiliations:** 1 National Engineering Laboratory for Tree Breeding, Key Laboratory of Genetics and Breeding in Forest Trees and Ornamental Plants, Ministry of Education, College of Biological Sciences and Technology, Beijing Forestry University, Beijing, China; 2 Kunming Botanical Garden, Kunming Institute of Botany, Chinese Academy of Sciences, Kunming, China; 3 National Tree Breeding Station for Platycladus orientalis in Jiaxian, Forest Farm of Jiaxian County, Henan, China; 4 Department of Ecology and Environmental Science, UPSC, Umeå University, Umeå, Sweden

## Abstract

*Platycladus orientalis*, a widespread conifer with long lifespan and significant adaptability. It is much used in reforestation in north China and commonly planted in central Asia. With the increasing demand for plantation forest in central to north China, breeding programs are progressively established for this species. Efficient use of breeding resources requires good understanding of the genetic value of the founder breeding materials. This study investigated the distribution of genetic variation in 192 elite trees collected for the breeding program for the central range of the species. We developed first set of 27 polymorphic EST-derived SSR loci for the species from transcriptome/genome data. After examination of amplification quality, 10 loci were used to evaluate the genetic variation in the breeding population. We found moderate genetic diversity (average *H*_*e*_ = 0.348) and low population differentiation (*Fst *= 0.011). Extensive admixture and no significant geographic population structure characterized this set of collections. Our analyses of the diversity and population structure are important steps toward a long-term sustainable deployment of the species and provide valuable genetic information for conservation and breeding applications.

*Platycladus orientalis* (L.) Franco, a member of the family, Cupressaceae, is one of the dominant forest species in China. It is native to northwestern China, Korea, and the Russian Far East, with a range from the south of Mongolia to the south-central China, with also a limited suitable area in Tibetan Plateau^1^,^2^. It is also now naturalized as an introduced species in Japan and India. This species is characterized with salt, drought and barren-tolerance, wide adaptability and strong resistance. Its roots can increase apparent soil cohension at a slope scale such as Loess Plateau, thus playing significant roles in windbreak and sand stabilization^3^. Consequently, it is commonly used for ecological restoration projects in arid mountain landscapes of northern China, where reforestation projects are extensively applied for sand stabilization and soil erosion control in the past decades. As a popular urban tree in cities in the northern China, it has remarkable ability to absorb and accumulate the atmospheric pollutants (SO_2_, Cl_2_) and heavy metal pollutants (Cu, Zn, As, Hg, Pb, Cd and Cr) in soil^4^. In addition, the woods are markedly decay-resistant and have outstanding resistance to humidity, making it valuable in building, furniture, and ship construction. It produces secondary metabolites that have insecticidal or biological activity against insects^5^. Because of these values, the demand for planting this high quality trees is increasing.

Started in the 1980 s, tree improvement and breeding activities of *P. orientalis* were established through provenance testing, phenotypic selection, and seed orchard establishment. Breeding populations were established based on extensive collection and testing of elite trees (plus trees) from range wide. The selected elite trees were collected into breeding archives and used to establish seed orchards for producing genetically improved seeds for plantation forests. Genetic basis of the breeding population decides the genetic quality and long-term potential of the breeding program and products. However, population genetic investigations in *P. orientalis* are thus very limited. Only one study based on allozyme markers^6^ and one study based on AFLP (amplified fragment length polymorphism) markers^7^ have been conducted on *P. orientalis* provenance trails and populations. These studies all revealed lower genetic diversity among the provenances of *P. orientalis*, and genetic diversity of the central populations is less than that of the south and the north regions of the distribution range. Whether this lower diversity in the central region would cause a problem for long-term breeding remains an important unanswered question. Understanding the genetic diversity and population structure is the necessary step in forming up strategies of genetic improvement and conservation, and is urgently needed for evaluating and adjusting the breeding program for *P. orientalis* in China.

Microsatellites, or simple sequence repeat (SSR) polymorphisms, are popular molecular markers for population genetic investigations. Carefully selected SSRs can behave as co-dominant genetic marker, and show high polymorphism. Despite growing competition from new markers such as SNP (single nucleotide polymorphism), these versatile and cost-effective SSRs will remain as a convenient tool in population and pedigree investigations of many plant systems. SSR development requires sequence data. The source of sequence data could be size-fractionated genomic DNA and EST (expressed sequence tag) library^8^ by the method of cloning, cDNA library construction and sequencing. Recently, high throughput sequencing techniques have allowed to identify sequences harbouring SSR motifs in non-model species^9^, which makes way for studying non-model plants like conifers with large and complex genomes. With increasing exploration of transcriptome data, EST-derived SSRs (EST-SSRs) have emerged as useful markers for estimating variation in coding sequences. EST-SSRs offer a number of clear benefits, including rapid and inexpensive development^10^. Also, EST-SSRs have been found to be significantly more transferable across taxonomic boundaries than genomic-SSRs^11^. To date there is no SSR systems available for use in genetic studies of *P. orientalis* and related species, which presents a handicap for potential genetic management and conservation for this valued conifer tree.

In this study, we first aimed to establish a set of promising SSRs for genetic diversity and mating system investigations. We then applied these markers to characterize the distribution of genetic variation in one important breeding population from the central range of *P. orientalis*. We wanted to understand the genetic property of this breeding stock and whether they can support the long-term breeding program for this species in central China.

## Results

### SSR development and polymorphism

We searched the transcriptome assembly of *P. orientalis*^9^ and the draft genome assembly of *Juniperus communis*^12^ for simple sequence repeats. From more than 1600 SSR loci, we selected and tested 292 loci, of which 108 yielded fragments of expected sizes, while the remaining ones failed to yield any products. Upon capillary electrophoresis, 87 loci produced clear and reproducible allelic patterns. Upon testing these loci in our samples, 60 turned out as monomorphic or very low polymorphism and 27 as polymorphic loci (Supplementary Table S1). The target sequences of these 27 loci are presented in Supplementary Data S1. After further testing, we kept 10 loci for genotyping of the 192 trees in the breeding population.

### Genetic diversity

**Figure 1:**
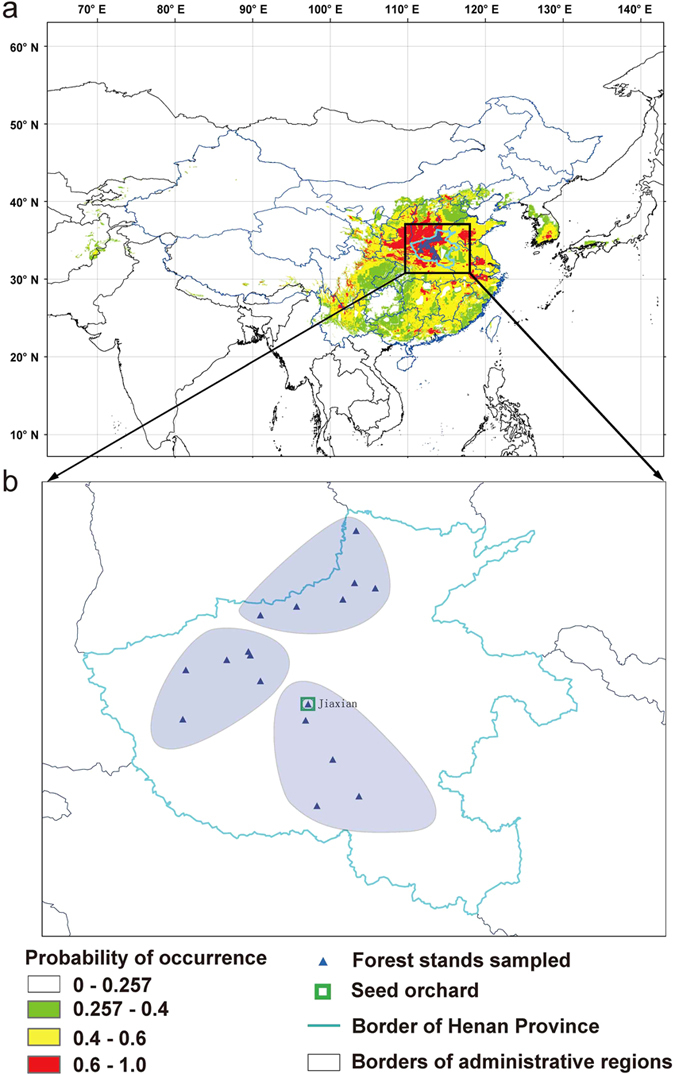
Geographic origins of the sampled elite trees. (**a**) Predicated suitable area of *P. orientalis*^2^ and the origins of our samples. (**b**) The composition and location of the 3 sampled populations. (Figure created in ArcGIS 10.2 http://www.esri.com/, and then modified with Inkscape https://inkscape.org/en/).

A total of 44 alleles were detected at the 10 SSRs loci in the 192 sampled elite trees (origins shown in Fig. 1), with the number of alleles per locus ranged from 2 to 7, and an average of 4.4 alleles per locus (Table 1). Expected heterozygosity (*H*_*e*_) ranged from 0.032 at locus Po108 to 0.573 at locus Po75, averaging 0.348 over all loci. Observed heterozygosity (*H*_*o*_) ranged from 0.033 at locus Po108 to 0.484 at locus Po75, averaging 0.280 over all loci. The estimated null allele frequencies (*P*_*n*_) ranged from 0.000 at Po107, Po108 and Po180 to 0.149 at Po173; 8 of the 10 analyzed loci had low null allele frequency, but two loci (Po145 and Po173) had *P*_*n*_ values close to 15%. Compared to genomic sequence derived SSRs in conifers (e.g. Norway spruce^13^), our EST-SSRs showed much lower allelic polymorphism. The statistical confidence for individual identification using the 10 loci employed in this study was moderate (*P*_*ID *_= 0.010). Thus not every tree had a unique multi-locus genotype; 14 genotypes each was assigned to 2–3 individual trees and a total of 29 trees had shared genotypes.

Based on their origins, the 192 trees were roughly divided into three populations, north, east and southwest. At population level, 14 private alleles (*N*_*p*_) were found at 7 loci distributed in the three populations (Table 2), with the frequencies ranged from 0.004 to 0.094. The number of different alleles (*N*_*t*_) revealed in each population ranged from 26 (the east population) to 40 (the north population). The effective number of alleles (*N*_*ea*_) and allelic richness (*A*_*R*_) estimated for each population ranged from 1.59 (the north population) to 1.83 (the east population) and from 2.47 (the north population) to 2.66 (the southwest population), respectively. The highest *H*_*e*_ estimate (0.393) occurred in the east population, whereas the lowest *H*_*e*_ of 0.333 was identified for the north population. The *H*_*o*_ in these populations followed the same pattern with the lowest value (0.258) found in the north population and the highest in the east (0.366), and all *H*_*o*_ values were lower than *H*_*e*_.

The single-locus tests for Hardy-Weinberg Equilibrium (HWE) in the three populations showed significant (*P *< 0.05) departure in 16 out of 30 cases, distributed over all 10 loci. All the departures were due to homozygote excess. The average value of inbreeding coefficient (*F*) over loci and populations was 0.197 and ranged from 0.070 in the east population to 0.225 in the north population (Table 2). The *F* values were significantly positive in the southwest and north populations. The excess of homozygotes at each locus was closely related to the frequency of null alleles detected at the locus (Table 1). We removed the two loci with high null allele frequency (po145 and po173) and recalculated the index. In this case, the average of inbreeding coefficient (*F*^*#*^) decreased to 0.126 (Table 2).

### Geographic structure

The estimation of genetic differentiation among the populations across the 10 SSR loci was very low (*Fst *= 0.011) (Table 1) translating to a high level of historical gene flow (mean *N*_*m *_= 22.48 migrants per generation). Pairwise population *Fst* values were very similar (0.011 to 0.012), and all the population pairs had significant gene flow (Table 3). AMOVA analysis produced similar result suggesting only 1.25% of the total variance were explained by differences among populations, and the rest were due to differences among individuals within populations and within individuals (Table 4).

**Figure 2:**
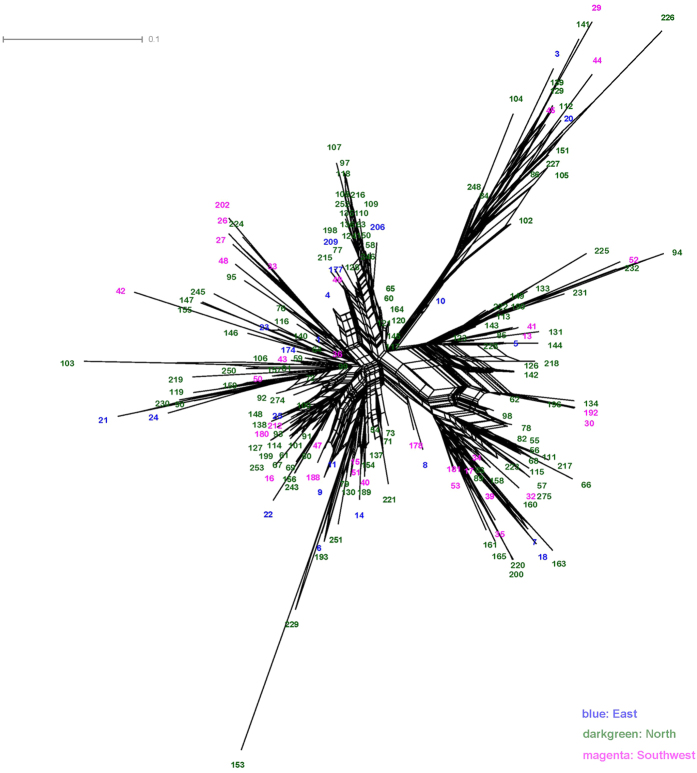
Neighbour network of *P. orientalis* individuals. NeighbourNet presenting the genetic relationship between individuals of *P. orientalis* as calculated by SplitsTree 4. Cross connections denote probable reticulation events between individuals like hybridization, or horizontal gene transfer.

**Figure 3:**
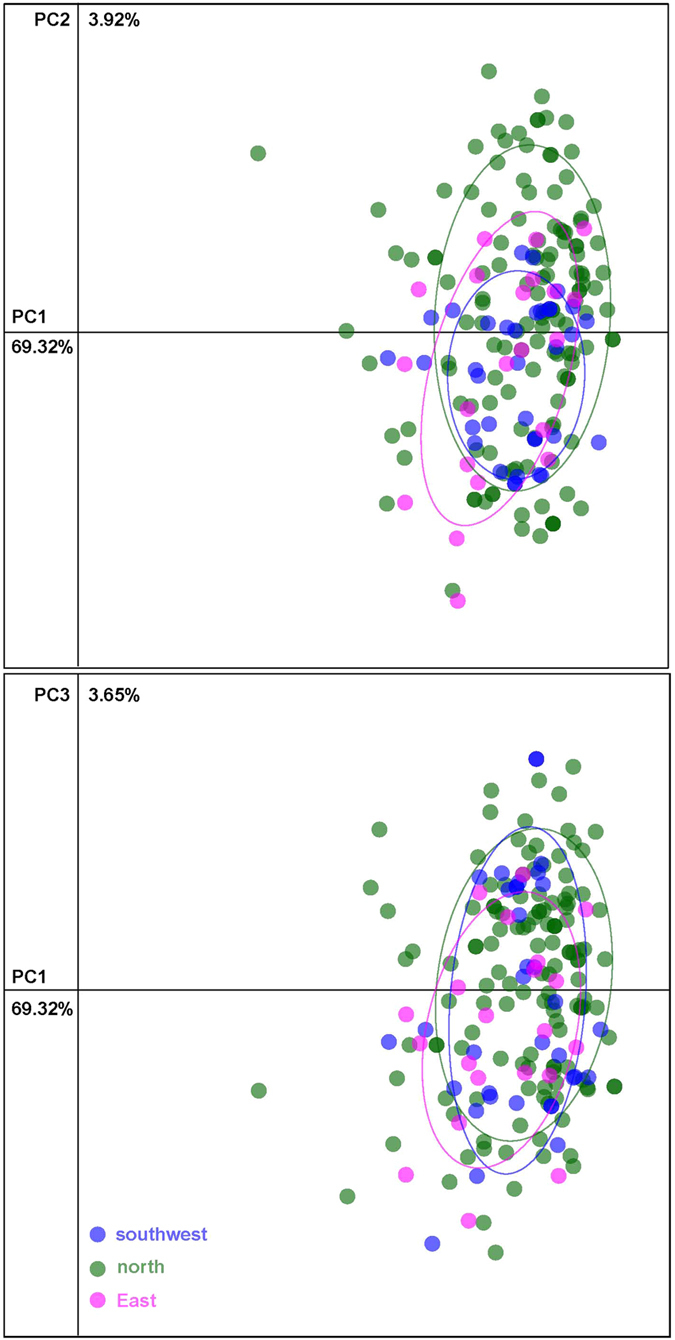
Principal Component Analysis (PCA) of *P. orientalis* individuals using genetic distance matrices. Individuals from 3 populations are indicated by the symbols illustrated. *P. orientalis.* Each point represents one genotypes; the ellipse-line with the same color stands for the boundary of the individuals from the same population source.

The neighbour network based on genetic distances among the 192 trees formed a big mixed network with no clear geographic structure (Fig. 2). The NJ tree for these individuals resembled that of the neighbour network (Supplementary Figure S1). Again, all the sampled individual trees were extensively mixed with each other and shared large amounts of recent admixtures, showing no clear pattern related to their origins. PCA partitioned 69.32%, 3.92% and 3.65% of variance in the experimental data to the first three axes, respectively (Fig. 3). Collectively, 76.89% of variation is explained by the first three components. The PCA results concurred with the network analysis, and showed no clearly separation of the populations into discrete clusters.

**Figure 4:**
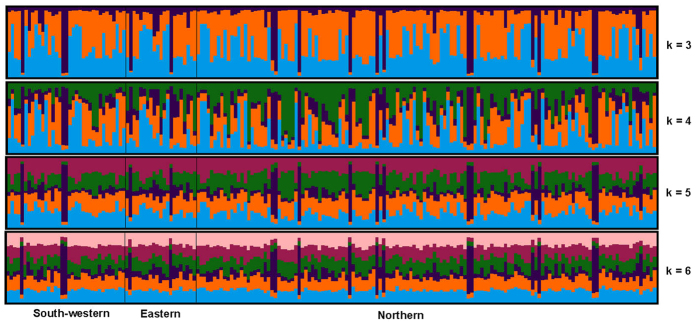
Admixture bar plots representing the identity of individuals based on assignment using Bayesian modeling. Each individual is shown as a vertical line partitioned into K colored segments whose length is proportional to the individual coefficients of membership in K = 3 to K = 6 genetic clusters that represent the populations assessed.

Bayesian methods implemented in the STRUCTURE^14^ revealed extensive admixed ancestry for each sampled *P. orientalis* individual and failed to reveal any meaningful genetic structure and geographical grouping for the three populations (Fig. 4). For each pre-defined population, we did not find any one dominant genetic cluster. Different genetic clusters expressed similar probabilities of occurrence in different population and no individual possessed coefficients of ancestry of 100% from one cluster, indicating extensive migration and gene flow.

Bottleneck tests conducted under the sensitive algorithm of IAM (infinite allele model) (Supplementary Table S2) indicated that the east population departed from the mutation-drift equilibrium which might denote evidence of a recent bottleneck in this area. Under SMM (stepwise mutation model), no population was detected to have a significant excess of gene diversity as compared with the gene diversity expected at the mutation-drift equilibrium.

## Discussion

*P. orientalis* is a widespread coniferous tree species with important economic and ecological values in China and other parts of Asia. The species’ wide adaptability and strong resistance increased its utilization, thus making it a suitable choice for both restoration and breeding activities. To establish a sound strategy for breeding and deployment, a good understanding of the distribution of genetic resources in the species range and the breeding population is a necessary first step towards a long-term sustainable goal. Up to now, genetic studies in *P. orientalis* are very limited, and available tools for genetic and breeding applications are under developed. For these reasons, we performed extensive data mining from the transcriptome and genome assembly of *P. orientalis* and a closely related species *J. communis* (also from Cupressaceae) for SSR markers that can be used for genetic diversity and breeding applications.

SSRs are widely used in many fields of genetic and breeding studies, such as assessing the distribution of genetic variability, mating systems, and parentage analyses in breeding and production populations^15^,^16^,^17^. However, no SSR marker systems have been developed for *P. orientalis*. Based on the transcriptome and genomic data, we successfully amplified 87 EST-SSR loci in *P. orientalis*, but only 27 loci showed polymorphism in our 192 trees. Of these 27 loci, 20 were developed from *P. orientalis* transcriptome data and 7 from the genomic data of *J. communis. Juniperus* and *Platycladus* are sister clades in the Cupressaceae family^18^. Inter-genus transferability of EST-SSRs seems reasonably successful in this study. Conifer genomes are large and complex with high contents of repeated DNA sequences, which make it difficult to identify unique locus that segregates in Mendelian fashion. Consequently, only a small fraction of SSRs selected from genomic databases can be converted to informative SSR markers. Nevertheless, SSRs derived from transcriptome database have obvious advantages over other strategies. The EST-SSRs can be rapidly developed from databases at a low cost, and EST-SSRs show a higher level of transferability to closely related species than genomic SSR markers^19^,^20^. The recent years saw a great accumulation of transcriptome sequence data for diverse plant lineages, following the application of high-throughput sequencing techniques. Mining the transcriptome data is an efficient way for marker development and offers great flexibility in selecting markers for different applications at different resolutions.

SSR fingerprinting technique provides an effective way in confirming genetic identities of accessions, parents, and their putative offspring. Mislabeling in achieves occurs often seed orchards^21^,^22^,^23^, and mislabeling may result in biased estimates of seed crops genetic quality. In the present study, 29 trees had shared genotypes over the 10 SSR loci. These matched pairs may present highly related sibs or mislabeling of the sampled trees during clonal propagation (grafting). Given the moderate power of discrimination of the 10 SSR loci employed, we cannot confidently discriminate the two alternatives.

This study represents the first investigation into the genetic composition of the founder populations of the *P. orientalis* breeding program in China. We investigated the levels and partitioning of genetic variation in the breeding stock for the central range of the species and found reasonably high variability and very low geographic differentiation in this collection.

An earlier study on this species using AFLPs detected low levels of genetic variability (the mean Nei’s gene diversity was 0.124) in 18 stands collected throughout the country^7^. Our study focused on the central distribution of the species, and found relatively high variability (*H*_*e *_= 0.349) in the 192 trees from three populations. Higher diversity is a desired property in germplasm resources to support a long-term effective breeding program.

We found very low population differentiation in the central range of the species; both *Fst* and AMOVA showed that only about 1% of observed total differentiations were partitioned among populations. Additionally, STRUCTURE, PCA and Neighbornet analyses all showed little geographic boundaries in the collection, and all trees had a mixed ancestry. The low divergence in the upland and the plain of central China could be due to several factors such as the relatively small geographic scale of our survey, continuous distribution, homogenous landscape, extensive gene flow, the population history and past anthropogenic activities. Gene flow promotes higher levels of genetic variation within populations and lower differentiation among populations which counteracts negative effects of genetic drift or directional selection on differentiation^24^. Conifers and other wind-pollinated tree may spread a part of their pollen over long distances. The effective pollen dispersal can extend up to 100 km in many tree species^25^. Like other conifers, *P. orientalis* is wind pollinated with high pollen production, both pollen and seed dispersal can be substantial. The studied trees were collected within a 230 km radius of Henan province where landscape is homogenous. The relatively small geographic range and plain landscape would see more frequent gene exchange and result in little differentiation between groups. However, pollination dynamics and mating system of the species remain to be poorly investigated, and should be a topic of research effort in the following studies to better understand the distribution of genetic variation.

Past anthropogenic activities can insert significant influence on the present population structure and patterns of genetic diversity in forest trees^26^. *P. orientalis* is a favorite temple tree of Taoists, Buddhist and Confucian priests and in many areas inside and outside China it has escaped from cultivation and established spontaneous stands^27^. Given these facts, the admixed ancestry and obscured geographic population boundaries could also be attributed to the anthropogenic influence, as Henan where the surveyed populations originated is a region with long history of human settlement. A range-wide population genetic study will be helpful in understanding the full picture of demographic history and genetic relationships among populations in this species.

Knowledge of the genetic structure of founder populations and patterns of gene flow is essential for establishing efficient breeding and seed orchard programs of conifer trees^13^. The selected trees from the central range of the species likely represent a considerable proportion of the available local genetic diversity. Moderate genetic diversity and extensive admixture characterized the elite breeding materials surveyed. To ensure that maximum genetic diversity is captured, more selection may be needed to support future breeding programs of the species.

## Materials and Methods

### Plant materials and genomic DNA isolation

In the mid of 1980s’, seed orchard (in Jiaxian Henan, China) was constructed by cloning 268 elite trees selected from 3 provenances (here the northern, southwestern and eastern populations) in Henan province which is in the central range of *P. orientalis*. In the present study, leaves of one ramet from each of the 192 elite clones with outstanding seed output potential were sampled for DNA extraction. The information about the 3 sampled populations (in this study, trees from close forest stands were regarded as a population) is shown in Fig. 1 (details were shown in Supplementary Table S3). The 3 populations represent the central of the suitable area of *P. orientalis* and cover the northern to southern Henan province with latitudinal range from 33.33° (N) at east to 35.42° (N) at north, and the longitude range from 112.17° (E) at southwest to 113.51° (E) at north. Yellow river geographically divides the sampled populations into two groups, one in the north (the northern population) and the other group in the southwest (the southwestern population) and east (the eastern population). And the north population is located in the southeast foot of Taihang mountains, the important mountain range and geographical boundary for eastern China. Each population was represented by 21 to 136 individuals.

The genomic DNA was extracted by using the method of cetyltrimethylammonium bromide (CTAB)^28^. Briefly, 200 mg fresh leaf tissues were ground in a 1.5 ml centrifugal tube with liquid nitrogen for each sample and added 800 μl CTAB buffer to the homogenate. The samples were mixed briefly and incubated for one hour at 65 °C. A 24:1 mixture of chloroform: isoamylalcohol was added to the homogenate, vortexed to mix, and centrifuged at 12,000 rpm for 10 min at room temperature. The supernatant of each sample was transferred to a new 1.5 ml centrifugal tube and mixed with one volume of isopropanol and incubate for 20 min at −20 °C. And we discarded liquid and collected the DNA-containing pellet after centrifuging at 12,000 rpm for 10 min. The precipitate was washed twice in 500 μl cold 70% ethanol. The ethanol was then carefully removed, and the resulting DNA pellet at the bottom of the tube was left to dry overnight. Finally, DNA was eluted by adding 70 μl water. The concentration and purity of DNA were determined by Nano Drop 2000 (Thermo Scientific). And the moderate DNA samples were diluted to 20 ng·μl^−1^ for PCR amplification.

### SSR development and genotyping

Our SSR markers were developed from two sources. For the first resource, a total of 1,684 loci with simple sequence repeats were retrieved from the high-throughput RNA sequencing based transcriptome assembly of *P. orientalis*^9^, and 196 loci were selected for further evaluation in the present study. For the second source, we identified 1,615 loci from the draft genome assembly of *J. communis* and then 96 loci were selected for further evaluation. In total, primers for 292 SSR loci were synthesized by Sangon Biotech (China). Polymorphism of the SSR loci was evaluated with a sample set of one individual from each of eight natural populations: Lingshou (Hebei Province of China), Mianyang (Sichuan), Feicheng (Anhui), Beizhen (Liaoning), Baoji (Shanxi), Bameng (Inner Mongolia), Heshui (Gansu), Nanping (Fujian).

QDD version 3.1^29^, a microsatellite selection and primer design pipeline, has been used to identify microsatellite markers from the transcriptomic and genomic sequence datasets and design primers.

The synthesized primer pairs were validated by polymerase chain reactions (PCR) using M13-tail technique, where a 5′ M13-tailed forward primer combined with a fluorescent label (FAM, HEX, TAMRA, ROX)^30^. PCR amplification of each locus was conducted in 20 μL volumes with 10 μL 2 × Taq PCR master mix (Biomed Technologies, Beijing, China), 4 μL (4 pmol) fluorescent-dye-labeled M13 primer, 4 μL (4 pmol) mixed complementary forward and reverse primers, and 2 μL (20 ng) genomic DNA. Conditions for PCR amplification were performed as follows: 94 °C for 5 min; 28 cycles at 94 °C for 40 s, annealing at 55 °C for 40 s and elongation at 72 °C for 45 s; 10 cycles at 94 °C for 40 s, annealing at 53 °C for 40 s, elongation at 72 °C for 45 s, with a final extension at 72 °C for 10 min^31^. The PCR products were used to analyze the fragments with an ABI 3730 sequencer. SSR alleles were called with GeneMarker version 2.20 (SoftGenetics, State College, Pennsylvania, USA). To minimize systematic genotyping error, PCR reaction and capillary electrophoresis were done with unique machines for a unique SSR locus, and all peaks binning were manually checked.

### Data analysis

Allelic frequencies including the number of alleles, number of effective alleles (*N*_*e*_), observed heterozygosity (*H*_*o*_), expected heterozygosity (*H*_*e*_) for each SSR loci were generated in GenALEx version 6.5^32^. Null allele frequencies (*P*_*n*_) for each microsatellite locus and each population were estimated using FreeNA^33^. GeneCap version 1.4^34^ was employed to detect samples with identical genotypes and samples differ by one and two alleles. And in each population, the total number of alleles detected (*N*_*t*_), the average number of alleles per locus (*N*_*a*_), allelic richness (*A*_*R*_), the effective number of alleles (*N*_*ea*_), the number of private alleles by population (*N*_*p*_), the observed (*H*_*o*_) and expected (*H*_*e*_) heterozygosity, and inbreeding coefficient (*F*) were estimated by SPAGeDi^35^. Hardy-Weinberg Equilibrium (*HWE*) based on Markov chain iterations and analysis of molecular variance (AMOVA) partitioning the total genetic variation among and within population was tested with ARLEQUIN^36^. The average pair-wise level of genetic differentiation (*Fst*) between populations was determined using multi-locus comparisons in GenALEx based on 999 permutations. The *Fst* value was also calculated by containing or excluding null allele (ENA) in FreeNA to examine the genetic divergence among populations and the potential effect of null alleles on genetic differentiation. As the *Fst* statistic is an indirect measure of gene flow, inversely related to the effective migration rate, it was used in the following formula *N*_*m*_ = 0.25 (1 − *Fst*)/*Fst* to estimate the number of migrants per generation between populations.

Recent population history was inferred by examining the departure from drift-mutation equilibrium based on allele frequencies using the software BOTTLENECK version 1.2.02 for each population^37^. The significance of potential bottleneck was estimated using one-tailed Wilcoxon sign rank test for heterozygosity excess, under infinite allele model (IAM) and the stepwise mutation model (SMM).

POPULATIONS software version 1.2.34 (http://bioinformatics.org/~tryphon/populations/) was applied to calculate the genetic distances among individual trees with distance of Dm, and Dm is considered the minimum genetic distance^38^. The NeighborNet tree was constructed and displayed by SplitsTree^39^. NeighborNet is similar to the common Neighbor joining method, but by showing reticulations it can represent alternative trees in the presence of distinct phylogenetic signals, which may arise, for instance, from gene flow between populations. Neighbour Joining was constructed and displayed by SplitsTree4^40^.

As an alternative approach for summarizing microsatellite variation across trees and populations, we performed principle components analysis (PCA) using the “adegenet” package^41^ in R^42^. PCA entails no assumptions regarding the cause of structure and, in contrast to other clustering approaches (i.e., STRUCTURE^14^), does not assume Hardy–Weinberg or gametic equilibrium.

In population structure analysis, the genetic structure of the 3 populations was analyzed using STRUCTURE version 2.3^14^, which assigns individuals to a number K of genetically homogeneous groups, based on the Bayesian estimate in accordance to the expected Hardy-Weinberg equilibrium and absence of linkage disequilibrium between the loci analyzed in each population. For the analyses with the program STRUCTURE, a burn-in period of 100,000 iterations and a posterior number of Markov Chain Monte Carlo (MCMC) of 200,000 iterations for K equals 1–11. For each possible value of K, ten runs were performed. Two different approaches were used to detect the most likely K value: the first was proposed by Pritchard *et al*.^14^ and based on the rate of change of LnP(D) for each K between 1 and 11 and the second was the criterion proposed by Evanno^43^, which is based on the second order rate of change of the likelihood function with respect to K (∆K) (the ad hoc ∆K test). The results from STRUCTURE were processed with the software STRUCTURE HARVEST^44^, which implements the Evanno method to identify the optimal groups (K). CLUMPAK^45^ was used to visualize the barplot of the probability of membership from the results of Q-matrix.

## Additional Information

**How to cite this article**: Jin, Y. *et al*. Genetic evaluation of the breeding population of a valuable reforestation conifer *Platycladus orientalis* (Cupressaceae). *Sci. Rep.* **6**, 34821; doi: 10.1038/srep34821 (2016).

## Supplementary Material

Supplementary Information

## Figures and Tables

**Table 1:** Characterization of 10 microsatellite loci isolated from 192 individuals of *P. orientalis.*

Locus	Allele Size Range (bp)	No. alleles	*N*_*e*_	*H*_*o*_	*H*_*e*_	*Fis*	*Fst*	*Fst (ENA)*	*P*_*n*_
po107	151–176	3	1.049	0.047	0.046	−0.017	0.008	0.008	0.000
po108	262–264	2	1.033	0.033	0.032	−0.014	−0.006	−0.007	0.000
po135	204–220	5	1.577	0.265	0.366	0.278^**^	0.011	0.004	0.088
po145	196–200	5	1.819	0.299	0.450	0.339^**^	−0.011	−0.009	0.135
po171	193–211	7	1.620	0.302	0.383	0.214^**^	0.031	0.026	0.079
po173	161–185	2	1.774	0.232	0.436	0.469^**^	−0.008	−0.005	0.149
po180	132–162	4	1.339	0.261	0.253	−0.027	−0.012	−0.009	0.000
po182	200–215	5	1.761	0.437	0.432	−0.007	0.070	0.064	0.025
po75	134–168	7	2.341	0.484	0.573	0.159^*^	0.000	−0.001	0.055
po53	206–212	4	2.014	0.443	0.504	0.124	0.029	0.029	0.038
All loci	—	4.4	1.633	0.280	0.348	0.197^**^	0.011	0.010	0.057

*N*_*e *_= number of effective alleles; *H*_*o *_= observed heterozygosity; *H*_*e *_= expected heterozygosity; *Fis *= inbreeding coefficient, computed as in Weir and Cockerham (1984), significant for ^**^p < 0.001; ^*^p < 0.01 based on 2000 permutations; *Fst *= genetic divergence among populations, computed as in Weir and Cockerham (1984); *Fst* (*ENA*) = *Fst* using the excluding null allele (*ENA*) method by Chapuis and Estoup (2007); *P*_*n *_= null allele frequency.

**Table 2:** Summary of genetic measures for the 192 individuals of *P. orientalis* divided into three populations.

Population	*N*	*N_t_*	*N_a_*	*N_p_*	*N*_*ea*_	*A*_*R*_	*H*_*o*_	*H*_*e*_	*F*	*F*^*#*^
North	136	40	4.0	12	1.59	2.47	0.258	0.333	0.225^**^	0.152^**^
Southwest	35	31	3.1	1	1.71	2.66	0.310	0.369	0.160^*^	−0.007
East	21	26	2.6	1	1.83	2.52	0.366	0.393	0.070	0.084
All	192	─	3.2	14	1.64	2.52	0.280	0.349	0.197^**^	0.126^**^

*N *= No. of individuals; *N*_*t *_= No. of different alleles (total) revealed in particular population; *N*_*a *_= No. of alleles per locus; *N*_*p *_= No. of private alleles; *N*_*ea*_ = No. of effective alleles per locus; *A*_*R*_ = allelic richness per locus; *H*_*o *_= observed heterozygosity; *H*_*e*_ = expected heterozygosity; *F *= nbreeding coefficient (significant for **p < 0.001; *p < 0.01); *F*^*# *^= inbreeding coefficient calculated after exclusion of two loci with the highest frequency of null alleles (po145, po173).

**Table 3:** Pairwise *Fst* (below diagonal) and *N*_*m*_ (above diagonal) values between the three populations.

	North	Southwest	East
North	—	20.583	20.583
Southwest	0.012	—	22.477
East	0.012	0.011	—

**Table 4:** Results of the hierarchical analysis of molecular variance AMOVA for 3 populations of *P. orientalis*.

Source of variation	df	SSD	Percentage of variation (%)
Among populations	2	4.02	1.25
within populations	189	201.06	23.58
Within individuals	192	125.50	75.16

Percentage of the total genetic variance due to each level and the probability test after 10,000 permutations. Degrees of freedom (df), sums of square deviations (SSD).
