# Supplementary material for: Genetic evaluation of the breeding population of a valuable reforestation conifer Platycladus orientalis (Cupressaceae)
Source: Sci Rep. 2016 Oct 10;6:34821. doi: 10.1038/srep34821 (PMC5056456; doi:10.1038/srep34821)
Supplement: Supplementary Information [file srep34821-s1.pdf]

Genetic evaluation of the breeding population of a valuable reforestation conifer *Platycladus orientalis* (Cupressaceae)

Yuqing Jin[1#], Yongpeng Ma[2#], Shun Wang[3], Xian-Ge Hu[1], Li-Sha Huang[1], Yue Li[1], Xiao-Ru Wang[1,4], Jian-Feng Mao[1*]

[1] National Engineering Laboratory for Tree Breeding, Key Laboratory of Genetics and Breeding in Forest Trees and Ornamental Plants, Ministry of Education, College of Biological Sciences and Technology, Beijing Forestry University, Beijing, China.

[2] Kunming Botanical Garden, Kunming Institute of Botany, Chinese Academy of Sciences, Kunming, China.

[3] National Tree Breeding Station for *Platycladus orientalis* in Jiaxian, Forest Farm of Jiaxian County, Henan, China.

[4] Department of Ecology and Environmental Science, UPSC, Umeå University, Umeå, Sweden.

[#] These authors contributed equally to this paper

[*]**Correspondence to:** Dr. Jian-Feng Mao

E-mail: jianfeng.mao@bjfu.edu.cn

Supplementary information

Supplementary Table S1 Characteristics of 87 polymorphic and monomorphic EST-SSR loci developed for *P. orientalis.*

Supplementary Table S2 Results of Bottleneck test for the populations under IAM and SMM models.

Supplementary Table S3 Origins of the *P. orientalis* samples.

Supplementary Data S1 The target sequences of the 27 polymorphic loci for *P. orientalis.*

Supplementary Figure S1 Neighbour joining tree. Phlylogenetic relationship of *P. orientalis* individuals.

Supplementary Table S1. Characteristics of 87 polymorphic and monomorphic EST-SSR loci developed for *P. orientalis.*

Locus	Primer sequence (5'-3')	Repeat motif	Allele size (bp)	Functional annotation	Tm (˚C)	HW
**Po53**	F: GTCGGGAGTTCTTGAACGAG<br>R: AAACTCTCATCCCTTCTTGGG	(AG)$_8$	186	no hit	54	ns
**Po57**	F: ATGGCTTTATTGCATCCGTC<br>R: ACAGAACATAGCATTAAACACCCA	(AC)$_7$	213	no hit	54	ns
**Po73**	F: TTGTGTCCCAGTTCAGTGGA<br>R: ATAACTGGCCATCCAGCATC	(AT)$_8$	284	no hit	54	ns
**Po75**	F: TGAAGCTTTCTTGAGGTAAGGG<br>R: TGAAGAGGCATAATACCGGG	(AC)$_8$	142	no hit	54	*
**jc52**	F: TCAATTGTCATGGGTCTAGGAA<br>R: GGTATCAAAGAACTTGGTGCTTG	(AG)$_6$	213	no hit	54	*
**jc38**	F: CGATGGCATCTTTGACCAAT<br>R: TTTCTTTGAAGTGTGATCGGG	(AAAG)$_5$	289	no hit	54	**
**Po45**	F: GCCTAACCGGAGGTATAGTTTG<br>R: CACTGCGTGGAAAGACTTCA	(AG)$_9$	103	no hit	54	ns
**jc89**	F: ATCCGGCCAAATAACACAAC<br>R: TGAGCCTCCTTCTTTCTTGC	(AC)$_5$	230	no hit	54	ns
**jc90**	F: GCTCGGCTTCTGAAGGACT<br>R: CACCTTCTGCCATCCCTTTA	(AAG)$_5$	332	no hit	54	ns
**jc10**	F: TTCTGTGTCACCTCTGTGGC<br>R: GACGACAAGGAGTGGTGGTT	(AG)$_5$	146	PMT	54	ns
**jc20**	F: GCATTCATGGACACATACAAGC<br>R: TGAACAAATGAAGGCATGGA	(AC)$_5$	236	no hit	54	ns
**jc69**	F: AGGACATTGAGAAGGCAACC<br>R: TATGCCCAAGGTAAAGGAGC	(AG)$_5$	297	no hit	54	*
**Po165**	F: CCGACACAAACTGTGTGCTT<br>R: AATGGCAGAATGACAATCCC	(AG)$_5$	170	Gp37_Gp68	54	ns
**Po156**	F: CCCAGAACCTCAAACCTTCC<br>R: TTCCGCTATACAACAATACGACA	(AG)$_5$	187	no hit	54	**

**Po103**	F: TGGCATAACCTACTTGCATCC<br>R: CTGTAAATGATAAATTAGGAAAGCTG	$(AT)_8$	275	no hit	54	*
**Po182**	F: TCTTGCTCTCAAACCATCCC<br>R: CCCATCATTCCCAATACCAA	$(AAG)_5$	191	DEC-1_N	54	ns
**Po161**	F: AATTTGTGCGCTTGAGCTTT<br>R: TGTGGCTTTAATCTGAGCTTG	$(AG)_5$	118	p450	54	ns
**Po171**	F: CTCACTTCCTGCCATAAGCC<br>R: CAAGTTGAACAGCCTCAGCA	$(AAAC)_5$	184	Mmp37	54	ns
**Po159**	F: TCCCGATAGTAAAGGAGCCC<br>R: TGTTTAGCGTCAGAAGAAGCAA	$(AT)_6$	200	no hit	54	ns
**Po107**	F: TGAGAGTAACAGCAGCAGGG<br>R: TAGGCCATTGTTTGAGATGG	$(AC)_5$	160	no hit	54	*
**Po108**	F: ATCATTCTCACTTGTGTCCAGC<br>R: GGGAAGAGGCTAACTTCGCA	$(AG)_5$	244	no hit	54	ns
**Po145**	F: AAAGCAAGCATGAGAACCAA<br>R: TCTCCCTCACTCCATCTTCC	$(AG)_5$	182	no hit	54	**
**Po114**	F: CTCCTGGATATTTATGCAATCG<br>R: TATTGCCTTGTGGTGAGTCG	$(AT)_5$	153	no hit	54	ns
**Po152**	F: AGATGGAAAGGATATTCCCAGA<br>R: ACATCTTCTCCCGACTTCCA	$(AAG)_5$	139	no hit	54	ns
**Po135**	F: GCTTCGAATTTGCTAGCCC<br>R: GCTCAAATCAGTTATCCTTGCAG	$(AT)_5$	190	no hit	54	ns
**Po173**	F: TTGAAGGAATGATCAAGGCTG<br>R: CAGACAAAGAACCACCACCA	$(AAC)_5$	161	no hit	54	ns
**Po180**	F: CCCTTTCTCTGAGCAAACCA<br>R: GGACTGGAGCTATTTGCCTG	$(AGC)_6$	126	no hit	54	ns
**jc1**	F: AAAGTTCCATATCTGGCGGA<br>R: TGGTATTCTTGGTATTCTTCTTCG	AAG	281	—	59	Monomorphic
**jc14**	F: TGAGTAGATGAGTGCGGGTG<br>R: GGTTCTCACCATCTTCGAGC	AG	292	—	59	Monomorphic
**jc15**	F: AGCAGACATCGATGAAGGTTT	AC	284	—	59	Monomorphic

	R: GCCAATAAATTGCATCTCCC					
**jc25**	F: GTGCATGACCTCATTGATCG<br>R: ACTCATTGTTTGGGCTTTGG	AC	161	—	59	Monomorphic
**jc26**	F: CTCCACCGATCAGATCAAGC<br>R: GGGAGAACCCTAAGGCCC	CG	244	—	59	Monomorphic
**jc27**	F: GTCCATTGAGTGTTGAACCAAG<br>R: GGTAGCTATGCCTCCAACCA	AT	255	—	59	Monomorphic
**jc30**	F: TGCAAACAATTTGGTAACGG<br>R: TGATACTTGGGAGAACTTGGG	AG	246	—	59	Monomorphic
**jc37**	F: CGAGGATATTGCAGACGGAT<br>R: TTCCACCAGGAGTAGCCTTC	AG	299	—	59	Monomorphic
**jc40**	F: CACAATGATCTCCTTTCATTTCC<br>R: AGGTAACTACAAGATCGCGAGG	AT	170	—	59	Monomorphic
**jc44**	F: TGGTTTCCCTTGAGTGCTTT<br>R: CCATTGTTGAAGATTTAATTGGTG	AG	294	—	59	Monomorphic
**jc46**	F: ATCCAAATGCGTTGTTCCAT<br>R: CCAGAGCAGCTGTAGGCAAT	AC	274	—	60	Monomorphic
**jc49**	F: GATGATTGATTACCCAAAGCAA<br>R: TTGGAGGATATGTGGAGGAAA	AAG	242	—	59	Monomorphic
**jc53**	F: GCTGATTGGATCTCAAGCCT<br>R: GGGTATTGAGTGCATGGAGG	AG	185	—	59	Monomorphic
**jc64**	F: AATTGGGAAAGCCTCAAAGC<br>R: TTGCATCCAAATAATCCCTTG	AC	171	—	59	Monomorphic
**jc65**	F: TGAAACTTTCTTGAGACCTCCC<br>R: GTTGTTACTGCCTCTTGCCC	AG	250	—	59	Monomorphic
**jc76**	F: GCACTTGAATTGAAGCCACA<br>R: CAACACCCTCAACACCACC	AAG	282	—	59	Monomorphic
**jc80**	F: CCAGAAACTCCTTCAGTACCTCA<br>R: ATGGAGAATGGAGGATGTGC	AT	299	—	59	Monomorphic
**jc82**	F: TTGTCATGGAGAACTTGGGAG<br>R: TCCAACCCATCAGACTATCCA	AT	292	—	60	Monomorphic

**jc84**	F: ATTTCACTGCTCTGTGGAGGA<br>R: AATAAGGCCAATGCTGATTCAT	AAT	341	—	59	Monomorphic
**jc91**	F: CTCATTCCAAGGCTCCAGAA<br>R: GCTCAGAAACCTCTCACCCA	AG	327	—	60	Monomorphic
**Po6**	F: TTCCAGTAATGATCCGTGGAG<br>R: CGTGGCAATACAAGACACAAA	AC	170	—	59	Monomorphic
**Po17**	F: GCGTTTGGATGTAAACTTGGA<br>R: AGAACCTGAGTTCGTGTGGG	AG	113	—	59	Monomorphic
**Po52**	F: GTGAATCTTGCATCAGCAGC<br>R: TTCACATGAAAGAGGCATGG	AT	229	—	59	Monomorphic
**Po62**	F: TGCCTTATGGTAAAGAACCCA<br>R: TGTTGACATCTCAGGCTCAAA	AT	212	—	59	Monomorphic
**Po80**	F: GCTGTGTTTCGCTTTACCGT<br>R: ACAGGAGTCAAGATCAGTGAAGG	AT	269	—	59	Monomorphic
**Po87**	F: GAGCAGTGGTACTGATTCCTTT<br>R: GTAAACCCGTTCATGTGCCT	AT	249	—	58	Monomorphic
**Po30**	F: GAGTTTATTGCCTCCCACAAA<br>R: ATTACGCATGCCCACAATTT	AG	126	—	59	Monomorphic
**Po38**	F: ATCTCACCCGATCAAAGCTC<br>R: ATTGTGTAGCGTTACCAAGGG	AT	194	—	59	Monomorphic
**Po78**	F: CTTATGCTTTACCTAACCATGCG<br>R: CAATCCTTTACTTGTAAGCGGC	AT	240	—	60	Monomorphic
**Po68**	F: AAATGCATTCAAACCAAGCC<br>R: AAGACGAAGACTCTGGTGCAG	AT	216	—	59	Monomorphic
**Po101**	F: CCCTCCCTCCTCATATAATCTC<br>R: AGGTAGACATAGAGAATGAATTCAAGA	AG	336	—	58	Monomorphic
**Po104**	F: AATAGGTTGGTGGTTGGTCG<br>R: GGCATAGCACCTCTAGCCC	AT	139	—	59	Monomorphic
**Po109**	F: GGTCTACTGCTATCCTTCACCC<br>R: ACAATGAGAAAGGTCCGCAT	AG	217	—	59	Monomorphic
**Po113**	F: GTTGATTTGCGGCTCAGG	AAC	118	—	60	Monomorphic

	R: CTCATCAAGTCCTCCCTCCA					
**Po117**	F: GTAGCAGGTAGCGCGGGT	ACT	191	—	59	Monomorphic
	R: CCTCGACTCTCGTCTTCGAC					
**Po162**	F: GCCCTTTAAACCCAATGCTT	CCG	133	—	60	Monomorphic
	R: CGGCGGAGTAAAGTGGTG					
**Po166**	F: GAGCAATCCACACCACCTTC	AAC	167	—	59	Monomorphic
	R: TGGATCATTGGTCTTCCCTT					
**Po168**	F: GGATCATGGGAGAATCGAGA	AC	125	—	59	Monomorphic
	R: TGCACTACTCCTTCGGTGG					
**Po177**	F: GTTGGCTGTATTGGGTTTGG	AGG	225	—	59	Monomorphic
	R: CAGCACTCAACTGATCGGAA					
**Po178**	F: AAGAAATGCCCTCTCAACGA	ATC	204	—	59	Monomorphic
	R: GGTTTGAAGTGTATGGCGGA					
**Po184**	F: TTCTCTCCCTCCACAACCAA	AG	140	—	59	Monomorphic
	R: CTCACATGCTGCTGTGGTTT					
**Po185**	F: CTTTCAGTCGCTCCTGCTTC	AT	139	—	60	Monomorphic
	R: CGGTTCCGTTTCATTAGCAT					
**Po186**	F: TTTGGAGAGTTTCTGGGCTG	AC	192	—	60	Monomorphic
	R: CTTCCCTACACTGCACCTGG					
**Po189**	F: TGTTTCCATCCTACCCTTGC	AG	126	—	60	Monomorphic
	R: CATTGGCTTTCCTACCTGGA					
**Po138**	F: GATTCTATGACGACAGCGGC	AG	115	—	60	Monomorphic
	R: TGATGGGTGGTATGATTCTCG					
**Po140**	F: CTTCTTGGTGATGTCAGAGGG	AAG	185	—	59	Monomorphic
	R: GAAGGAAGAGGAAGCCAAGG					
**Po141**	F: GAATTCAAGGAACCATGGGA	AG	181	—	59	Monomorphic
	R: TCCAATGTTGACCAAAGAAGG					
**Po142**	F: CCCAACTATCTCCAGCTTCG	AT	224	—	59	Monomorphic
	R: ATTAGTTGTGGTGGCAGGCT					
**Po143**	F: TAGCCTTTGGGCTTATCTGC	AG	239	—	59	Monomorphic
	R: AAAGCATCTTCCCTGTGAGC					

**Po146**	F: CTCCGTATACAAGTGGCGGT<br>R: TTGCATCCGTTAGTATTCCAAA	AG	127	—	59	Monomorphic
**Po147**	F: ATGGCATCCTCCTCTTTCCT<br>R: GAAAGGCCACAAAGAAACCA	AG	239	—	60	Monomorphic
**Po148**	F: GTTCTTTCCTACCAGCGCC<br>R: CAGACCCAGAAATTGTTTCCA	AT	187	—	59	Monomorphic
**Po150**	F: AGTTCAGATTTGTCCAACACGA<br>R: CGCAATTGTAAGGGTTCCAG	AC	153	—	59	Monomorphic
**Po151**	F: TTCCCAATCCGTAATACCCA<br>R: AATGCAACCAGGAAGTAGGG	AG	177	—	59	Monomorphic
**Po153**	F: ATCCGACGACCTTCTGAGC<br>R: AACACGCTTGACTTCCCTTG	AGG	178	—	60	Monomorphic
**Po154**	F: ATTGCTTCCGTACAAGGCAC<br>R: TGAGAGGCTCACAAGTTACCG	AT	164	—	60	Monomorphic
**Po155**	F: GGATTGTTTGGTTTCATTTGC<br>R: ACCAAGTTGCTAGAGACGCC	AG	170	—	59	Monomorphic
**Po157**	F: TATCCCGGAGAACAAATTGC<br>R: CAAGTGACCATGAGTGGAGC	AG	176	—	59	Monomorphic
**Po158**	F: AAAGAAAGCGAAGAGAGGGC<br>R: CAGTGTGCTTCTTCTGCCTG	AG	173	—	59	Monomorphic
**Po102**	F: TCTCATAAAGGTACAATGCATACACA<br>R: TACAGCAGCGTAGTCCCATC	AT	350	—	59	Monomorphic

The locus names with "Po" indicate the locus developed from the transcriptome assembly of *P. orientalis*; the locus names with "jc" indicate the locus developed from the draft genome assembly of *Juniperus communis*. Protein family annotation are prepared by querying the protein family database, Pfam[1]; HW: ns = not significant, * $p < 0.05$, ** $p < 0.01$, *** $p < 0.001$.

1 RD, F. *et al.* The Pfam protein families database. *Nucleic Acids Research* 28, 263-266(264) (2000).

Supplementary Table S2. Results of bottleneck test in the 3 populations under IAM and SMM models.

Population	IAM	SMM
North	0.784	0.997
Southwest	0.278	0.839
East	**0.014**	0.326

IAM: infinite allele model; SMM: stepwise mutation model. The significant value was indicated in bold type.

Supplementary Table S3. Origins of the *P. orientalis* samples.

Population	Forest stands	Sample Size	Longitude (E)	Latitude (N)	Accessions
North	Linxian, Huixian, Weihui, Huojia, Boai, Jiyuan	136	113.51º	35.42º	54-69, 71-73, 76-86, 89-95, 97-124, 126, 131, 133-151, 153-161, 163-165, 189, 190, 193, 196, 198-200, 215, 232, 43, 245, 246, 248, 250-253, 274, 275
East	Jiaxian, Xiangxian, Wugang, Leshanlinchang, Biyang	21	113.43º	33.33º	1, 3-11, 18, 20-25, 174, 177, 206, 209
Southwest	Yiyang, Luoning, Luanchuan, Luoyang, Longmen, Linru	35	112.17º	34.36º	13-17, 26, 27, 29, 30, 32-36, 39-45, 47-53, 178, 180, 181, 188, 192, 202, 212

Supplementary Data S1

>Po53
AGGGTGTATTTGTTACAGTTCAATAATTTGCCATAGAGAAGAACCAATTCGTTTGAGGATAGAGTGTGCTCCCTATATTCATCTATAGGAGGATG
TGAAGCTTGATCCTTGTCGGGAGTTCTTGAACGAGGAGATCAAAGTTAGGGCCCCTTTCTTATTAGGTTGGTGTCTTCCATGAACAATGGTGCC
CCTCCTCCAGActctctctctctctctACGTCATTGGCATTGTACAACAATCAAATACTTGGGAAATCTGAAGTGGATAGATACTACCCAAGAAGGGATGA
GAGTTTATCTATTTTGTCCATTTTGTCTTTGGCTGGCTGAAAGCCATATAATTCTCTTACAATGGCCCTCTGTGAGAGGGTTGCTAATCTTTTCCA
TCATGGATAAAATGGTGATAATACTAGGAT

>Po57
TagagagagGGTTTATAGTTTAGCAATCGGAAAGCTAAGGTATAGTGTTGTGAGGAGGtttttttttGAGAGTAAGGGTTTCTAGTTTAGCACTCAGAAAG
CTAAGGTATAGGGTTGTGAGGAGGTAttttttGAGAGTATCGCTGATAAGATGTGCTATCCATGTTCCAGGATGGTAAAAtttttGATAAGGTGTTCTATG
GCTTTATTGCATCCGTCTCCGGAATAATTTTCATCTGCATCGGTTTTATTGAATTTGGAATaaaaaaCgtgtgtCGTTCTTAATGTCGTTTTGGGTCAGGA
ATATTAATTTGTCTCTGGTTATCGCttttttAAAATTGTAACCGGCTTAGTTTTGTcacacacacacacatatataGTTTAATTTTGGGTGTTTAATGCTATGTTCTG
TCT

>Po73
ATaaaaaGGAAGTAACGATAGACTTTGTTGTGATTCTATTACTTCCAAAATATTATTTCGAGTGGTATCTTTTGTTGGTTGTGTCCCAGTTCAGTGGA
AAACATTAATTGTTAGTATTGTACTGGTGATTACTTGAAATCTCCCACAGAAATCACTTTTAGGATGGAGACTTGTTAAAACATaaaaaaTACAAGT
TGGTTGGTTATGCTTGTTTACCATCAAAGGTAGGAATCACCACACtatatatatatatataGCTTCAGTTTAAAAGAATGTCAATGAGTCAATACCATCTAG
TGAGCTATGTACCTCAGTTTAAACAGGATTTTAttttttCCGGTTGATGCTGGATGGCCAGTTATGTAATGAACACAAGTTATCAAATCATTTGCATTG
AAACGCTGTTAAAATGTTTT

>Po75
CTGAAGCTTTCTTGAGGTAAGGGGTTTTACTCCTGTAAATAGTTGTAATATAATTTTGTTGTGCTTGAtttttGgtgtgtgtgtgtgtgtGCCCAGTGGCTCCTC
TGctctctGCAAGCGACTCCCGGTATTATGCCTCTTCATGACTTTATGCCCTATGGATAAAGCACTGATCCACTTTCTGAGTTCTGGCATTCCCTTTAT
TCAGAGCCTATGTTTTCCTTTAAATAAGGTTGCAACTGTTTCCCCTTTTAAATTTGGTTTTCAGTTCCAGAGATTTGCATGATCAAAGTTCCCAA
CCAGTCTAAAGCCCAATTACTCCGCTTTCCGGTAAGCAAATCTGATAGGGTCCAAcccccTATGCCCGAGTCATACCATTCAAGCAAATTCCTGAT
ATTGAATAAACCAGTTTGACC

>Jc52
GACTACACTACTCGTAATAATATCTCCTTCTTTCATGCTCATTTCAACAAAATTTCCTGTCTAGCTTTCAACTTTAGCCCTTAACACAtttttGTCTCC
TCAACAAATTTGTGCCAACTTATCCCATATCTTCTTTGTAGTAGTGTAATCCTTCACATCATAAAACTTAGCACACTTAACTTAAGGAAGATGCCA
AATCTATCTTCATTTTATTGTGTTCCTGCTTCTCTTTTATTTTCTCAATTGTCATGGGTCTAGGAAATTCTATGTATTCATTGTCTAAATAGTATAGTA
GAGATTCTCCAACTTTAGATAGATGCAATCTCACTAGATGTTTCCATTGTAGGATATTTTCTTTGTTCAACTTGGCTACctctctctctctTGAATATCTTT
ATCTTTAGCTTAATTCCCTTCAAAATTTCCTTACAAGCACCAAGTTCTTTGATACCAATTGTTAGAATGATTAGACTAAC

>Jc38

GACTACACTACTCGTATGTCTTCACAAGGTTGAACAGCGACCTCTAtttttGCGATGGCATCTTTGACCAATAGTATGTTGgagagaAGTTAGTATGAG
TGTAATGGGATGAATGTAGTCAAGAAGTTGTCATATCAATCAAACTCTTAGGTACAGATGATCAACTCCCTCAAATACCATCTATCAAAACAGCT
AAAGGAATCTTGGACATCTTGAAGGATCTTTACGAGATATCCATCAAGAttctttctttctttctttctATGACCAATTAGAGGCTATTGGCCGCAAGATAGAG
GAAGATTACAATGAAAAGCTTGCCCGATCACACTTCAAAGAAATGCCCAATAATATCACTTACACTAGTGTTGCCCTAACATTCTCTAATCTTTG
CAACAAGCTTCTACAGCAAGATAGATGGAAGGAACAGTTTGGAAGTTATG

>Po45
TAATGCCAAACTTGGTCAAAATGCCTAACCGGAGGTATAGTTTGTGATATGAGAAGCTATGCACAGCTCctctctctctctctctctCATCCCCATTGATGGC
CATGAAGTCTTTCCACGCAGTGATGGTGCCATTCCCTGTCCAgggggCAATCAATCCTTTCATGCAATTGGCCCACCTtctctcCGCAAGGGGATTCTT
CATCACCTTCGTTAACACTGAGTATTGCCAACGCCGCATGGTAGACGAGGCTGGACAAGCAGAGGGGTTCCGGtttttGACTGTCCCGGACGGCC
TACCGCCAGAGCATGGCCGTGAGCATGGCCGTGTCGATGATCTTGTCAACTATTTGATTGCACTGGAAAATCTAGCCCCTGTTTTGGAGAAGCA
TCTGCTTGCCATGACTCGTTCGGAGGA

>Jc89
GACTACACTACTCGTAAGGGTCAAAACCATGTCAAACAACAGAGGATAATAGACGAGGGGAGAAAAGAGTAACAACACCACCTTGACTAGCT
TCATGCTGAGAAGCaaaaaTGGTACTATCCGGCCAAATAACACAACAAGATGAGGTAAGAAGAAACCCAACTACTTTAACTTCCTAAAGGTAAA
GAACATCAAGATCAAATCAACATTTGGTATcacacacacaATATTTTCTAGGGAGGTCTGTCAGcccccATTCCATGACATGCAATTCATGGTAACCCAA
CTTGCATGTCATCCGCATCCCCAagagagGAGAAATTGTTATGCAAGAAAGAAGGAGGCTCAAAAGAGGGGTCATCAAAAGCTGTGACCTTTCA
AAGCTTCTCCCCATTATCaaaaaaCTCTTCTCGGCCATCTAGGCTACTAGACCAGCATAGCTTCTATTGGTCTATCATTGACACCACTTGCGGCTCTT
GGGTCCAAAGAGGAAGCAGATCTCGAGTATGAACAACCATTGTCCCCTGACCTCCAGGGTTTGAAACANGAGGCAAAGGGAGCACTGGGAA
CTGGGGCTTCATGGGGAACCTGACTTGAGCTAGATTTACCAT

>Jc90
GACTACACTACTCGTAACTTGAGCCTAGTGCTAAGATTTGCAGTGGTATAACTTTAGTGCTCGGCTTCTGAAGGACTTTTGTAGGAGAGCCAGA
TGTTGGTAGAGTTACCAATTCTTAAATGGCCTCTTATTCCTTCTCCTTTGGTATGTCCTTATTTTCTTCAGATCTACCTTGGTTAATCACCATGATCT
AACTTGTACCTTATGTTTGTACATCAAGTACAGATGCATCTATTTGTCCTATTTGTTCTTTTGTAGTCTTCtcttcttcttcttctTTCTTTTGTTTAATtatataAA
ATCTAACAATTGTGTCAACCTTTGTAGATGTATCTTCTAATGTTTGTATTAAAGGTTGAGCACCAATAGCTAAAGGGATGGCAGAAGGTGGTTCT
TGTGTATTCACCTTATCCCTTGTAATGTCAGCTACTACCTTCTAAAGTTCATCAACACTGACCAATATTGGATTTTcacacaATCTGGCTTGTAGTGT
GGATTGGTCAAGGATCTTCTTTAAGGttttttGAGTTT

>Jc10
GACTACACTACTCGTACTTACACCCGTTCACTATTTCCAGAACTTTCTGTCCATCCAGCGTCCATAACTTGATTGCTCCATTATTGCCTACTTCCT
GAATCTGATATGCCCCAACCCACTTAACTTTGAACTTTCCAGGTTTGATCTCATTCCAGCCGTCGTACTTCAACACCCATTGTCTAGGTTGGAAC
CTGTTCATTTTCAAATGCTTATCGTGCCATGCTTTTGCGCCTGTTCTGTGTCACCTCTGTGGCCCACTATGCCTATGATCGTCGAAGTTTATTTAA
GTTACACGGtctctctctcAAGTTTTCCATATCGCCCAATTTGTTCCCGATACTCGGTACCATGAATTTCGTTAACCACCACTCCTTGTCGTCAATAACT
GTATTCTTGATTTTAGAACCTTCACTCCGTATTGTTGCAGCTTCTATTAAACCCTTCCTCTTTGTTTCCTATGCAGATCGTATGATGTGTCTTCCTT
GCTGCCGG

>Jc20
GACTACACTACTCGTTAGATACATGAATCCAGATTTGTATGCGTGAATGTATGTATGCATACatatatTTCAATATGCACATGTTCATACATAAATACAT
AGATGAATGGATTTGCACatatatTCACTCATTCGATCatatatTCATAcattcattcattCAATCATACATTCATCCATACCTTCATACCTTCATACATACATCCAC
ACCTTTATTCATTCTCGCCTTCTTGCATTCATGGACACATACAAGCATTCATTCATACATACCTTCGTATCTTCATAAATGCATCAATATACACATTC
ATGCATTTATAATGTATTTGTATTTGTACATGCATGCATAGATACATGTAGACATGCATATAGCTACCTATCTATCTTTACATACATGAATCcacacacaca
TACTTCCATACATTCAGTCATCCTTGTATACACTTTTGCGTCCATGCCTTCATTTGTTCAGTCATTAATCCATACCTCCATACTTATGAACATTCAAT
CATACCTTCATTATTCATCCATACATTCATTCCTTATTCA

>Jc69
GACTACACTACTCGTTTGATGATTTACATGGGGCACCcttcttcttCAAAATTAATTTTCTATATGGATACCATCATATCCATATgagagaAGAGGACATTGA
GAAGGCAACCTTCTGATGTCACTATGGCCACTTTGAATTTATGGTTATGCCATTTGGGCTAACCAATGCACCCACCACATTTTAGAGTTGTATGA
ACTAGGTATTCAAGAGGCAATTGAGGAGATTCATCTTGATCTTCTGTAATGACATCTTGGTCTTAAGTAAGACATAGGAGGAGCACTTAGAGCA
TCTGGAGGAGGTGTTATCTATTCTagagagagagTTATTGTATGCCAAGGAATCTAAATGTGAATTTGGCATAACCAAGCTCCTTTACCTTGGGCATAT
CATTAGTGCAGATGGTGTTTGAGTTGATACTAAATAGATTAGAGCTATTTTAGATTCACCTACTCCCACAAACCTcacacaGCTTAAGAAGATCTTT
GGTTTATGTG

>Po165
GGAGTGaaaaaGAAGCCAGGCATTAGTTGGATTATGACCaaaaaTAAGGTGCATACTTTTGTGGCAGAAGACAGATCCCATCCTCAAACAGAGGAG
ATCTATGCAATGTTGTCTGctctctctAGaaaaaTGGAAGAGGTGGGTTATGTACCCGACACAAACtgtgtgCTTCATGAAATGGATGAGGACACGAAAGA
ACActctctctctTACCACAGTGAGAAACTTGCTATATCTTTTGGTCTTATTGCGACACCCGCAGGGACGCCCATCCATGTCATGAAGAACCTTCGTGT
TTGTGGGGATTGTCATTCTGCCATTAAGTTCATATCTAAGATTGTTGGTCGAGAACTTGTTGTAAGGGATGCCAATCGTTTCCATCATTTTAACAG
TGGGCTCTGCTCGTGTGG

>Po156
ACCCAGAACCTCAAACCTTCCTTTAAACCTTCCTTTTGTGTTATaaaaaGACAATTCCATAAAGAATAATAGCATTAAATCAGTTCTTTTAAAATAG
ACATagagagagagCAGCAAATACAAATCAAGGAGCTGAGAATTTCTTATACTCCTCGCAAAACTAGATGTCGTATTGTTGTATAGCGGAAAACACC
AAATCCATGTACACAGT

>Po103
AATGTTTTGGATCACACTCTATGACTCTATGATCCATCATCAGAACAAGAGTGAAAGGAAACATTAAAGTACTGAAATATGAAATATGAAATGC
AAATTGGGACAGTAAAGTTGGCATAACCTACTTGCATCCATGCTTTTGGAAGCATTTAATTCCCATACTTAGGGAAATATTGAGGCTCAGAAAAT
CATATTATGAGAGTTGGATTTAAAGTAAAAGCaaaaaCAATCAATTCACCTTCCATTCCAAAAGAAACAAACAGATTTTCTATCTGCTGTTCCATTT
AATAatatatatatatatatGGTCATAATGAATGTCCTTGTTTCtttttCTCAACAAAACACGAATaaaaaTTACAGCTTTCCTAATTTATCATTTACAGCAATTA

>Po182
TCCGGGAAAGCACTAGCGGTTACGACACCGTCCATTCAAGCCATCTTTACGTCATTACGACCTCCTCAAATCACatatatTCTTGCTCTCAAACCAT

```
CCCAGTGCAATTATTTCCTCTGCTTTACGCGCTGTTTGATTTTGTTTATTTCTCGGCAAAGGAAATTAAAAGCCTCAAGCTTTTAGATTGTTTTAG
GTCCAGGgaagaagaagaagaaGTTATGGCTAGAATCAATGAAGTTATGGCCATTCTTTTGGTATTGGGAATGATGGGGCATTCCACTGCGCAGGCTCC
TTTAGGTTCTCCTGTGGCATCGCCCACAACGTCGCCGGTTCCAACCCCGGCAAAGACACCCAcccccTCTGCACCTCCAGCGTCCTCACCTTCAC
CTGTTGCTTCCTCCCCTGCAGTTGCACC

>Po161
GGATCATACTGGAGAAATACCAGaaaaaTTTGTGCGCTTGAGCttttttCTTCTAAACAGCTCCAAACATTCCGGCCCAagagagagagGGAGATTTGTAAG
ACTCTTAGTATTCTGTTTCAACAAGCTCAGATTAAAGCCACAGTTAATATGAGAATTGTTTTGTCTCAGATGACATTAAATGTTATGATGAGCATG
ATAATGGGCGATCAGTGGTTTGTTGAGGAagagagCATGTCCTGCGCAGAAATTTTACATCTGATTGAAGAGTCTTTCATGCTCCATGGgtgtgtCAAT
GCTGGAGATTATATTCCCCTGCTGAAGTGGTTTGATTTGCAAGGGT

>Po171
GGCTCCTCGTTGTTGACAATGCAACCTGGCTGAGGGAAAACTCCAGATCCATATGCACACGCAAATTCAACAGGCGGCATTTGTTTAAGAGGT
TCCGCTAATTCACAACTAAGCTCTTCGCCTGACTTAGAAAACTCACTTCCTGCCATAAGCCCTAAACCCTAGTAACGCCGATACCCTGGGACAA
ATCTTCTAACAAGcaaacaaacaaacaaacaaaCTATTTTCGTTGATTTAACATGTTACTTCTAGTGTCATCTGAACCAAGCAGCTAAGATAGCACTACGAT
GGAGAAAACTGCTGAGGCTGTTCAACTTGTTTCGGGCACTTCGGTAAATCGTAACACTGTCGATAATAGGTACTAGTCGCCTGGACATCATTGA
TCAGAAGTTCGATTTTCACTGCTATTAGAATTGGCAA

>Po159
ATGAGTTGTCTCCCGATAGTAAAGGAGCCCGATCATAATGAAGGCTACCGATAATCAATGAAACTTGATCACAGTATTTGCTGATAATCCATATAA
TATTGCAAGGATGTAGGCatatatatatatGCTTATATCCATGCGATTTATGGAATCAGCAGTAATTTCCAGTATATCAAGTAATTCATGTTTTGCTTCTTC
TGACGCTAAACATTAACACTCCCTCTTTAGCTAGGAAGAAGGaaaaaaaCATCAATAGGTCAGAATCATCATATAATCAACACAATATGAACAGTAC
ATGGTATATCCAATGATCT

>Po107
TGTGCAACAAATTCAAAAGtttttCTTGCAGATGAGTGacacacAAGTCAAGTCCTGCAAGAAATCTCACAAGATTGACGTGAGTTTCAAAAGCTAA
CCTGGAAAATTTCAAGCAAAGACTTGGCTACTGAGAGTAAcagcagcagGGGTTTCAACACCttttttCCGCCATTTTGTCTTTGGCAGGTGCAATCAG
AGCAGATTGGAAACATacacacacacATACGCATGCACGCACACGcacacacaTCCCCTTTGTTGTCCCCTGCCATCTCAAACAATGGCCTAAAACTCAC
ATCCCCAAAACC

>Po108
GGTTAACAGGACCGATACAATCAGAACAAATAGATTCATTTTGACTACTTCTGGTATAAGCTTAAatatatCTCAAGCCAGTGATACTCTTAAATTAT
TTCTGCACTTGCACTCAGTtatataTCCTCTAGttttttCTGGTTATAGGTTCATGGATATCATTCTCACTTGTGTCCAGCTACCTTGCCCTGTGGTTCAAT
TAAGGTGGTCTTACAGATTTCTAATGGCAGAGGACATACCTGATTTTAAGTTATGTCGGTGCTTTTAAGAGTAAtctctctctcATAAGGTTAAATTCGG
AGATATTTACTCATTGTCAAGACACTAGCAAAAGTTGATTGTATGAATTAAATAGTTCATCGGAAGCATCTAAATGACAGATGCGAAGTTAGCCT
CTTCCCCGAACTAGC
```

>Po145
AAAGCAAGCATGAGAACCAAAATAAAGGTGACTCTaaaaaCACaaaaaGGAGAAGAAATAAGAAGCAAATAAATATGAAagagagagagATAAACTAA
AATAAGAGaaaaaTGAAAACCCAAagagagagaaaaaagggggAGAGGGGAGGAGCACAAGAAGGAAGATGGAGTGAGGgagagaCAACATAGaaaaaCAA
AGAAACTAGAgggggg

>Po114
CGCAGTTCTTGATCATCCCGATAATCAGTGACGaaaaaTGGCTCGGAATGTGTCAAAGTATTAAATGAATATCTTATTCttttttCCTAATTTACCTAAAG
CTCCTGGATATTTATGCAATCGTACACAtttttCTGGTTTTCCAAAAGGTCCtatatatataAGCAATCTTACTCAttttttCTAGttttttATAAGTTCCGAAatatatAA
GCATTATGGGGAGCATACAAGCGACTCACCACAAGGCAATATCAGTAATATCGCAGATACGGC

>Po152
GGCAATTAGGCTTTATAAGATGGAAAGGATATTCCCAGATTAGTAGACTGTTCTCAAACAGTGCATATGGAAGACATGGCCTGCaagaagaagaagaag
ATGATGATATAGAAGATGAAGtttttAAGGGCTTCCATGGAAGTCGGGAGAAGATGTCTAGTGAAATTGGCTATGGGGCTAGTAATGAGGATAAGT
TCGAATATGATTACGAGCATCCAAGAGTCAAGGAAGCTTGTAGGCTCATTAAATTGCAGCATATGTGGGAGACACCGCTTGAAAGGGACCTCA
GGCGCACGGTTCGCAAACTAACTCCTCA

>Po135
GTTTGCTTCGAATTTGCTAGCCCAAGTATGTATTTAAGGGCTGCATCAATATCAGCCCTACATCAAATATCGATGTCTACTtatataTCAGATTTTGCT
GatatatatatGCTTCAGTTTGCTTCCCTTGAATCGCACTTGTACTGGCTATTAATTAGGTGATGATAAGGACTGCAAGGATAACTGATTTGAGCATATT
GAATTTGCC

>Po173
CCTTGGGTAATTATTTtatatataatatatatACAAATCCCTGTAAGTGAGACAAAACTGAGTAATACAATACCACAGTTCTTGTTATGAAttttttGCATAAAAT
TTAAATTTATTGCTGATGCTTTATTAGAAGTTTGAAGGAATGATCAAGGCTGTTTCTCTGTGTTCTGATATAACTGCACTGTAAACTTGGTTTCttgtt
gttgttgttgAGTAtttttGTCagagagATaaaaaTAGCTGCTCACTTCCTTtatataTGTCAAACTGAGTAAtggtggtggTTCTTTGTCTGTCATTGCACTAtttttGTCACA
GCTACAGAACTAGttttttAACTATGCTTCCCCTAGTGTTTTGGGATGGGGATGGCAGGAGGCAATGATCATGTAAAAGAAAAGAAttttttttt

>Po180
TGCCATGGTACAAGCATTTATTTATAAAatatatGAAGTCCTGCTACTGCAGCTTCTAAGTAGTACTACCTTTAGCTAAGAAAGAGGATTAATAATAG
AGTCCAAATGGGCTCAGCCTTTTATCCCCACCATTGATGCACTGCAACACCCCTTTCTCTGAGCAAACCATAGAGTGCCAGGCACTCCCAGTAT
GATCTCCTctgctgctgctgctgctgCTTCTGGTAATGGCATTCAAGGAAGAGTTCATCAATCAGGCAAATAGCTCCAGTCCTCAGCATTTTAGGCAATATC
TGAAATTCAGCTCCACCAACATCCATTTTCATGACAACATAGTCATCTGCATTCACAGTTTTCATTAACCAATCTGCCAAATCCAAAGACCTGAC
TCTTTTCTGCTCAATCTCTATTCCATTCT

Supplementary Figure S1. Neighbour joining tree.

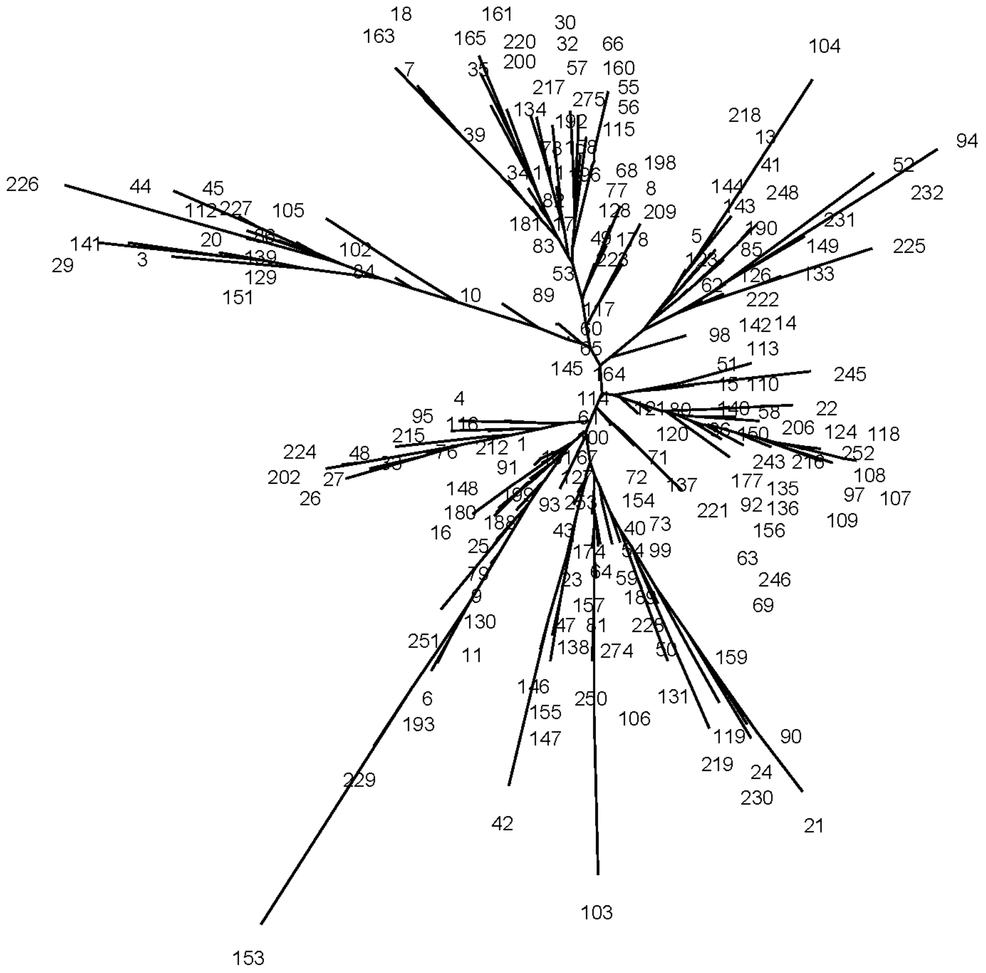

Phlylogenetic relationship of *P. orientalis* individuals.
